# Priority European strategies for sustainable access to high-quality genetic counselling in cancer: A Delphi study

**DOI:** 10.1038/s41431-026-02015-y

**Published:** 2026-02-13

**Authors:** J. Matt McCrary, Els Van Valckenborgh, Denis Horgan, Evgenia Aleksandrova, Ralf Bargou, Regina Lohajova Behulova, Ivica Belina, Ann Liza Egesberg Bøhme, Joan Brunet, Florin Burada, Adela Chirita-Emandi, Andrada Ciuca, Chrystelle Colas, Anastasia Constantinidou, Razvan-Ovidiu Curca, Viorica Cursaru, Miriam Dalmas, Zanda Daneberga, Evandro de Azambuja, Antoine De Pauw, Robin De Putter, Turem Delikurt-Tuncalp, Deirdre Donnelly, Hans Ehrencrona, Lenka Foretova, Fabrizia Galli, Maurizio Genuardi, Rachel Giles, Claire Grima, Ramūnas Janavičius, Helena Kääriäinen, Barbara Klink, Mateja Krajc, Joanna Kufel-Grabowska, Baiba Lace, Liis Leitsalu, Christophe Le Tourneau, Marianne Lodahl, Francesca Mari, Erika Matos, Luca Mazzarella, Tamara Hussong Milagre, Martin Mistrik, Barbara Moss, Amy Nolan, Rosie O’Shea, Milena Paneque, Attila Patócs, Rebecka Pestoff, Hélène A. Poirel, Martine Risch, Manuel Rodrigues, Katharina M. Roetzer, Andrea Ros, Evelin Schröck, Gunda Schwaninger, Lucie Slámová, Kostas Stamatopoulos, Sonja Strang-Karlsson, Krzysztof Szczałuba, Virginie Szymczak, Philippe Theis, Jacqueline Turner, Olga Valcina, Christopher Vella, Wendy A. G. van Zelst-Stams, Karin A. W. Wadt, Johannes Zschocke, Joelle Ronez, Tim Ripperger, Marc Van Den Bulcke, Anke Katharina Bergmann

**Affiliations:** 1 Institute of Clinical Genetics and Genomic Medicine, University Hospital of Würzburg, Würzburg, Germany; 2 Department of Human Genetics, Hannover Medical School, Hannover, Germany; 3 Cancer Center, Department of Epidemiology and Public Health, Sciensano, Brussels, Belgium; 4 European Alliance for Personalised Medicine, Brussels, Belgium; 5 Association of Cancer Patients and Friends - APOZ, Sofia, Bulgaria; 6 Comprehensive Cancer Center Mainfranken, University Hospital of Würzburg, Würzburg, Germany; 7 Department of Clinical Genetics, St. Elisabeth Cancer Institute, Bratislava, Slovakia; 8 Coalition of Associations in Healthcare (KUZ), Zagreb, Croatia; 9 From Testing to Targeted Treatments (FT3), Brussels, Belgium; 10 Department for Patient Support and Volunteering, Danish Cancer Society, Copenhagen, Denmark; 11 Hereditary Cancer Program, Catalan Institute of Oncology, IDIBGI, IDIBELL, Girona-Barcelona, Spain; 12 Department of Genetics, University of Medicine and Pharmacy of Craiova, 200349 Craiova, Romania; 13 Regional Centre of Medical Genetics Dolj, Emergency Clinical County Hospital Craiova, 200642 Craiova, Romania; 14 Department of Microscopic Morphology, Genetics Discipline, Center of Genomic Medicine, University of Medicine and Pharmacy “Victor Babes”, Timisoara, Romania; 15 Regional Center of Medical Genetics Timis, Clinical Emergency Hospital for Children “Louis Turcanu”, (part of ERN ITHACA), Timisoara, Romania; 16 Department of Psychology, Babeş-Bolyai University, Cluj, Romania; 17 Genetics Department, Institut Curie, Paris, France; 18 INSERM U1339, DNA Repair and Uveal Melanoma (D.R.U.M.), Paris, France; 19 Department of Oncology/Haematology, Medical School, University of Cyprus, Nicosia, Cyprus; 20 Elysee Hospital Alba Iulia, Oncology Department, Alba Iulia, Romania; 21 Myeloma Euronet Romania, Bucharest, Romania; 22 Office of the Chief Medical Officer, Department of Policy in Health, Ministry for Health and Active Ageing, Valletta, Malta; 23 Institute of Oncology and Molecular Genetics, Riga Stradins University, Riga, Latvia; 24 Institut Jules Bordet, Hôpital Universitaire de Bruxelles (H.U.B), Université Libre de Bruxelles (U.L.B), Department of Medical Oncology, Brussels, Belgium; 25 Paris Science & Letters Research University, Paris, France; 26 Center for Medical Genetics, Ghent University Hospital, Ghent, Belgium; 27 Department of Clinical Genetics and Genomics, The Cyprus Institute of Neurology and Genetics, Nicosia, Cyprus; 28 Northern Ireland Regional Genetics Centre, Belfast Health & Social Care Trust, Belfast, Northern Ireland; 29 Department of Clinical Genetics, Pathology and Molecular Diagnostics, Skåne University Hospital, Lund, Sweden; 30 Division of Clinical Genetics, Department of Laboratory Medicine, Lund University, Lund, Sweden; 31 Department of Cancer Epidemiology and Genetics, Masaryk Memorial Cancer Institute, Brno, Czech Republic; 32 aBRCAdabra ETS, Settimo Milanese, Milan, Italy; 33 Sezione di Medicina Genomica, Dipartimento di Scienze della Vita e Sanità Pubblica, Università Cattolica del Sacro Cuore, Rome, Italy; 34 UOC Genetica Medica, Dipartimento di Scienze di Laboratorio ed Ematologiche, Fondazione Policlinico Universitario A. Gemelli IRCCS, Rome, Italy; 35 VHL Europa, Dordrecht, The Netherlands; 36 Patvocates, Riemerling, Germany; 37 Department of Pathology, Mater Dei Hospital, Msida, Malta; 38 Faculty of Medicine, Department of Human and Medical Genetics, Institute of Biomedical Sciences, Vilnius University, Vilnius, Lithuania; 39 State Research Institute Center for Innovative Medicine, Vilnius, Lithuania; 40 Vilnius University Hospital Santara Klinikos (VULSK), Hematology, Oncology and Transfusion Medicine Center, Oncogenetic Unit, Vilnius, Lithuania; 41 Finnish Institute for Health and Welfare, Helsinki, Finland; 42 National Center of Genetics, Laboratoire National de Santé, Dudelange, Luxembourg; 43 MGZ - Medical Genetics Center Munich, Munich, Germany; 44 Department of Clinical Cancer Genetics, Institute of Oncology Ljubljana, Ljubljana, Slovenia; 45 Clinic of Oncology and Radiotherapy, Medical University of Gdańsk, Gdańsk, Poland; 46 Genetics and Rare Disease Center, Riga East Clinical University, Riga, Latvia; 47 Institute of Clinical and Preventive Medicine, University of Latvia, Riga, Latvia; 48 Estonian Genome Center, Institute of Genomics, University of Tartu, Tartu, Estonia; 49 Genetics and Personalized Medicine Clinic, Tartu University Hospital, Tartu, Estonia; 50 Department of Drug Development and Innovation (D3i), Institut Curie, Paris-Saclay University, Paris, France; 51 Department of Clinical Genetics, Copenhagen University Hospital Rigshospitalet, Copenhagen, Denmark; 52 Department of Medical, Surgical and Neurological Science, University of Siena, Siena, Italy; 53 University Hospital of Siena, Siena, Italy; 54 Department for Medical Oncology, Institute of Oncology Ljubljana, Ljubljana, Slovenia; 55 Laboratory of Translational Oncology, European Institute of Oncology IRCCS, Milan, Italy; 56 Associação EVITA - Cancro Hereditário, Lisbon, Portugal; 57 European Patient Advocacy Group, ERN GENTURIS, Bertinoro, Italy; 58 Department of Medical Genetics, Unilabs, Spišská Nová Ves Slovakia; 59 Inspire2Live, Amsterdam, The Netherlands; 60 Irish Cancer Society, Dublin, Ireland; 61 School of Medicine, Trinity College Dublin, Dublin, Ireland; 62 School of Medicine and Health, The University of Sydney, Sydney, Australia; 63 Cancer Genetics Service, St. James’s Hospital, Dublin, Ireland; 64 i3S - Institute for Research and Innovation in Health, University of Porto, Porto, Portugal; 65 IBMC - Institute of Molecular and Cellular Biology, University of Porto, Porto, Portugal; 66 CGPP - Centre for Predictive and Preventive Genetics, University of Porto, Porto, Portugal; 67 ICBAS – School of Medicine and Biomedical Sciences, University of Porto, Porto, Portugal; 68 Department of Clinical and Molecular Genetics, National Institute of Oncology, Comprehensive Cancer Center, Budapest, Hungary; 69 Department of Laboratory Medicine, Semmelweis University, Budapest, Hungary; 70 Clinical Genetics Department, University Hospital Linköping, Linköping, Sweden; 71 Institution of Biomedical and Clinical Sciences, Linköping University, Linköping, Sweden; 72 Service Psychosocial, Fondation Cancer, Dudelange, Luxembourg; 73 Department of Medical Oncology, Institut Curie, Paris, France; 74 Labdia Labordiagnostik, Vienna, Austria; 75 St. Anna Children’s Cancer Research Institute (CCRI), Vienna, Austria; 76 Department of Genetics, Hospital Universitari Germans Trias i Pujol, Catalonia, Spain; 77 Institute for Clinical Genetics, University Hospital Carl Gustav Carus at TUD Dresden, University of Technology and Faculty of Medicine of TUD Dresden, University of Technology, Dresden, Germany; 78 ERN GENTURIS, Hereditary Cancer Syndrome Center, Dresden, Germany; 79 National Center for Tumor Diseases (NCT), NCT/UCC Dresden, a partnership between German Cancer Research Center (DKFZ), Faculty of Medicine and University Hospital Carl Gustav Carus, TUD Dresden University of Technology and Helmholtz-Zentrum Dresden-Rossendorf (HZDR), Dresden, Germany; 80 German Cancer Consortium (DKTK), Dresden, Germany; 81 German Cancer Research Center (DKFZ), Heidelberg, Germany; 82 Institute for Human Genetics, Medical University of Innsbruck, Innsbruck, Austria; 83 Department of Genomics, Institute of Hematology and Blood Transfusion, Prague, Czech Republic; 84 Institute of Applied Biosciences, Centre for Research and Technology Hellas, Thessaloniki, Greece; 85 Department of Clinical Genetics, HUS Diagnostic Center, University of Helsinki and Helsinki University Hospital, Helsinki, Finland; 86 Center of Excellence for Rare and Undiagnosed Disorders and Department of Medical Genetics, Medical University of Warsaw, Warsaw, Poland; 87 Clinical Genetics Centre for Ophthalmology, The Mater Misericordiae University Hospital, Dublin, Ireland; 88 Latvian Association of Oncology Patient Organizations, OncoAlliance, Riga, Latvia; 89 Malta Health Network, Valletta, Malta; 90 Department of Human Genetics, Radboud University Medical Center, Nijmegen, The Netherlands; 91 Department of Clinical Genetics, Maastricht University Medical Center, Maastricht, The Netherlands; 92 Institute for Clinical Medicine, Copenhagen University, Copenhagen, Denmark

**Keywords:** Genetic counselling, Health policy

## Abstract

Europe’s Beating Cancer Plan is a substantial European Union (EU) investment into cancer prevention and treatment. Integration of genetic services towards personalised cancer prevention and care is a flagship of this plan. Genetic counselling is critical to this integration, facilitating informed patient decision making and improved clinical management. However, growing demands for genetic testing and concurrently increasing workforce shortages necessitate new strategies to equitably ensure sustainable access to counselling across the EU. This project aimed to inform future European activities by identifying priority European strategies for addressing common European genetic literacy, workforce, and reimbursement barriers to genetic counselling in cancer noted in prior work. A Delphi survey was conducted, with genetics, oncology, and patient stakeholders invited from all EU Member States. The response rate was 62% (124 total invitations). Over three phases, 77 participants – 28 geneticists; 14 oncologists; 18 genetic counsellors; 16 patient representatives; 1 otherwise qualified expert – rated 19 strategies according to their Importance, Urgency, and Feasibility and selected their top three priority strategies. Five strategies met pre-defined consensus thresholds and received a clear plurality of priority ratings: (1) EU-wide genetic counsellor recognition; (2) Including genetics expertise in oncology guideline creation; (3) Shared EU genetic counsellor registration/education with legal weight; (4) Mandatory counselling reimbursement when clinical guidelines are met; (5) Mandatory inclusion of genetics in oncology fellowship/continuing education. Results provide a roadmap of European actions which promise to sustainably improve access to genetic counselling in cancer care. Upcoming and ongoing EU projects promise to advance their implementation.

## Introduction

The inauguration of Europe’s Beating Cancer Plan (EBCP) in 2021 marked the beginning of a new era of European Union (EU) investment into coordinated activities to improve cancer prevention, diagnosis, treatment, and survivorship [1]. EBCP is organised around ten flagship initiatives, of which two relate specifically to genetic/genomic technologies: Flagship 6 ‘*Cancer Diagnostics and Treatments for All’* includes an aim to “improve access to innovative cancer diagnosis and treatments [including] ‘next generation sequencing’ for quick and efficient genetic profiles.”; and Flagship 7 ‘*The European Initiative to Understand Cancer (UNCAN.eu)’* includes an aim to facilitate personalised approaches to cancer prevention and care through increasing integration of polygenic risk scores [1]. The prominent feature of genetics in the EBCP underscores the growing importance of genetic technologies in guiding targeted cancer treatments and prevention strategies, in particular to address the estimated 5–15% of cancer cases linked to genetic cancer predisposition syndromes [2].

Funded under the EBCP, the CAN.HEAL project (https://canheal.eu) ran from November 2022 – April 2025 with the broad objective to ‘Build the EU Cancer and Public Health Genomics Platform’ and provide a critical foundation for the realisation of EBCP Flagships 6 and 7. Included amongst the activities of CAN.HEAL was a work program related to genetic counselling, in recognition of its essential and often legally mandated [3] role in helping patients and their families understand and make informed decisions about genetic testing and subsequent measures [4].

Ideally, genetic counselling is delivered by health practitioners with clinical/medical genetics training and expertise, including clinical/medical geneticist physicians and/or genetic counsellors, but also non-genetics physicians and health professionals with genetics competencies [3, 5]. Genetic counselling delivered by individuals with insufficient genetics expertise has been linked to negative impacts on care through avoidable costs (*e.g. unnecessary genetic tests*), suboptimal clinical management, and negative psychosocial outcomes in patients and family members [6].

An increasing demand for genetic testing related to the push towards personalised cancer medicine across paediatric and adult patient populations [7] is also leading to increasing demands for genetic counselling by appropriate experts. Rapid advances in knowledge and methods (*e.g. evolving gene panels, multi-omics analyses, polygenic risk scores*) further fuel needs for both broad and further specialised genetics expertise to meet clinical demands. However, shortages in both the general healthcare and clinical/medical genetics workforce across EU Member States already exist and are predicted to further grow over the coming decades [8, 9]. New strategies are needed to ensure that quality genetic counselling can be provided in sufficient volumes and in a reasonable timeframe to meet increasing genetic testing demands across the EU.

To guide the development of these new strategies, preliminary work identified common barriers to the delivery of high-quality genetic counselling present across Member States: genetic literacy (*of patients and non-genetics health professionals; a noted barrier in all 27 Member States*); workforce capacity (*a noted barrier in 25 Member States*); and insurance reimbursement (*a noted barrier in 13 Member States*) [3, 10]. These common barriers were identified in similar proportions by both expert health professionals and cancer patient organisation representatives [3, 10].

The three identified European barriers to genetic counselling in cancer provide clear targets for new strategies and indicate that European-level action is likely to most efficiently address these shared challenges. Accordingly, we aimed to identify, through structured consultation with key genetics, oncology, and patient stakeholders from across the EU, priority European strategies to address these three common European barriers to genetic counselling in cancer and guide EU policy and initiatives.

## Subjects and methods

### Participants

Invitations to participate in a Delphi survey were sent to 1–3 individuals from each of the following four stakeholder groups per Member State (*n* = 124 total individuals) by the lead author (JMM) in consultation with the corresponding author (AKB): clinical/medical geneticist physicians; clinical/medical oncologist physicians; genetic counsellors; and cancer patient organisation representatives. Invitations were sent to at least one clinical/medical geneticist physician, clinical/medical oncologist physician, and cancer patient organisation representative from all 27 Member States; at least one genetic counsellor (*specialised allied health professional, qualifications defined according to European Board of Medical Genetics registration criteria* [11]) was invited from the 15 Member States in which genetic counsellors are known to be active [12]. Individuals across stakeholder groups were invited because they were in a leadership position of their national genetics/oncology society or national patient organisation (defined according to membership in the Association of European Cancer Leagues) and/or recognised as possessing nationally-recognised expertise in genetic counselling in cancer (*e.g. through prior work with the CAN.HEAL consortium* [3, 10]). In some cases, multiple individuals from a given stakeholder group and Member State were invited to collectively achieve the desired combination of expertise and national leadership. This approach was favoured over a broader ‘snowball’ approach to participant recruitment because it avoided responses from individuals with unknown expertise and national representativeness and allowed us to quantify response rates.

### Delphi survey

A modified ‘Policy Delphi’ process was used to identify priority European strategies to address common barriers to genetic counselling in cancer [13]. Given the large number of invited stakeholders (*n* = 124), a structured approach was favoured in all three Phases described below, forgoing an initial phase of open-ended questions. All Phase I participants were invited to contribute to both subsequent Phases (II and III) [14]. Individual participant responses and identities were kept confidential throughout the Delphi process [13]. Thresholds for consensus ‘high’ Importance and Urgency ratings for a given strategy were defined a priori and as per prior Delphi studies as an average rating ≥7 out of 9 [13]. The following descriptors were defined to contextualise average Feasibility ratings: low feasibility (1–3); moderate feasibility (4–6); high feasibility (7–9).

#### Phase I

Participants were first asked to rate (1-9 Likert scale [13]) nine European strategies proposed by the research team (JMM, TR, JR, AKB)(Table 1) according to their ‘*Importance (i.e. impact potential)’, ‘Urgency (i.e. need for swift implementation)’*, and ‘*Feasibility (i.e. ease of implementation)’*.

**Table 1:** European strategies for addressing common barriers to genetic counselling proposed by the research team.

Strategy Number	Descriptive Text
P1	EU-wide recognition of genetic counsellors as allied health professionals and appropriately qualified experts to deliver genetic counselling.
P2	Establish an EU system of registration, education and accreditation for genetic counsellors (allied health professional) with legal weight in Member States.
P3	Establishment of target numbers of clinical/medical geneticist physicians per 100,000 population for Member States.
P4	Mandate full reimbursement of genetic counselling when conducted according to national or European guidelines.
P5	Mandate full reimbursement of genetic counselling regardless of delivery mode (e.g. remote, face-to-face) for both patients and clinicians/allied health professionals in each Member State.
P6	Mandate the availability of genetics continuing professional education for non-genetics health professionals in each Member State.
P7	Mandatory education for clinical oncologists – continuing and as a part of fellowship training – regarding screening for cancer predisposition syndromes (e.g. when to refer, how to screen).
P8	Development and EU-wide implementation of a best-practice approach to genetics education in secondary students.
P9	Mandatory inclusion of genetics expertise in the creation and revision of national/EU clinical oncology guidelines.

Participants had the option to provide additional text comments for each strategy, as well as propose and rate (*Importance, Urgency, and Feasibility criteria; 1-9 Likert scale*) up to three of their own strategies.

#### Phase II

Participants were presented with a summary of all Phase I ratings, and asked to rate 10 additional strategies (*Importance, Urgency, and Feasibility criteria; 1-9 Likert scale)* which were aggregated around common themes from 64 strategies proposed by participants in Phase I. Additionally, participants were asked to designate their #1, #2, and #3 priority strategies from the 19 strategies presented in Phase I and Phase II to help define a focused set of strategies to guide future action.

#### Phase III

To further refine the relative prioritisation of the highest ranked strategies from Phase II, participants were asked to again designate their #1, #2, and #3 priority strategies from five strategies which received a clear plurality of priority rankings in Phase II.

### Statistical analyses

Priority rankings were weighted using the Borda count developed for ranked choice voting (i.e. Priority #1 ranking = 3 points; Priority #2 ranking = 2 points; Priority #3 ranking = 1 point) [15]. The non-parametric Kruskal-Wallis 1-way ANOVA was used to test for significant differences in Importance, Urgency, and Feasibility ratings and weighted priority rankings between the four stakeholder groups: clinical/medical geneticists; clinical/medical oncologists; genetic counsellors; and cancer patient organisation representatives. When significant main effects were found, pairwise comparisons between professional groups were performed using the non-parametric Dunn’s test and Bonferroni correction for multiple comparisons. The Kruskal-Wallis 1-way ANOVA with post-hoc Dunn’s tests with Bonferroni correction were also used to test for significant differences in response rates across the four stakeholder groups and 27 EU Member States. Differences in weighted priority rankings between strategies were tested using the non-parametric Friedman test; pairwise post-hoc comparisons were conducted using Wilcoxon signed rank tests with the Bonferroni correction for multiple comparisons when significant main effects were observed. Significance was set at α = 0.05 for all statistical tests. All statistical analyses were performed in SPSS version 29.0 (IBM Corporation).

### Role of funding source

The European Commission had no role in the study design, collection/analysis/interpretation of data, or writing of the report, nor in the decision to submit the paper for publication.

## Results

A total of 77 individuals accepted invitations to participate in Phase I of the Delphi survey from 124 total invitations (62% overall response rate). Response rates for each stakeholder group were: clinical/medical geneticists (29 of 44, 66%); clinical/medical oncologists (13 of 33, 39%); genetic counsellors (18 of 20, 90%); and cancer patient organisations (16 of 27, 59%). A total of 34 participants were from Southern/Eastern European countries (44%), with 43 from Western/Northern European countries (56%), as classified by EuroVoc [16]. Response rates were significantly different across stakeholder groups (χ^2^(3) = 13.5; *p* = 0.01) but not EU Member States (χ^2^(26) = 19.2; *p* = 0.86). Pairwise post-hoc comparisons showed significant differences in response rates between clinical/medical oncologists and genetic counsellors (*p* = 0.004), but no other significant pairwise differences (*p* > 0.32). The majority of Phase I participants were aged 41–60 (*n* = 57; 74%) and female (*n* = 50; 65%). In Phases II and III, 66 and 68 individuals participated, respectively (86–88% retention rate). See Table 2 for a breakdown of participants by Member State, Phase, and professional group.

**Table 2:** Delphi survey participants, organised by Member State and professional stakeholder group. Individuals with both clinical genetics and clinical oncology qualifications were counted only as clinical geneticists (*i.e. their primary practicing specialty*).

MEMBER STATE	NUMBER OF PARTICIPANTS														
	*Clinical/medical Geneticist*			*Clinical/medical Oncologist*			*Genetic Counsellor*			*Cancer Patient Organisation*			*TOTAL*		
	*Phase*			*Phase*			*Phase*			*Phase*			*Phase*		
	*I*	*II*	*III*	*I*	*II*	*III*	*I*	*II*	*III*	*I*	*II*	*III*	*I*	*II*	*III*
***Austria***	2	2	2	-	-	-	1	1	1	-	-	-	3	3	3
***Belgium***	2	2	2	1	1	1	3	3	2	-	-	-	6	6	5
***Bulgaria***	-	-	-	-	-	-	=	=	=	1	1	1	1	1	1
***Croatia***	-	-	-	-	-	-	=	=	=	1	1	1	1	1	1
***Cyprus***	-	-	-	1	1	1	1	1	1	1	-	-	3	2	2
***Czech Republic***	2	2	2	-	-	-	=	=	=	-	-	-	2	2	2
***Denmark***	2	1	1	-	-	-	1	1	1	1	-	1	4	2	3
***Estonia***	-	-	-	-	-	-	1	1	1	-	-	-	1	1	1
***Finland***	2	2	2	-	-	-	=	=	=	1	1	-	3	3	2
***France***	1	1	1	2	2	2	1	1	1	-	-	-	4	4	4
***Germany***	2	1	2	1	1	1	1	1	1	-	-	-	4	3	4
***Greece***	-	-	-	1	1	1	-	-	-	1	1	-	2	2	1
***Hungary***	2	1	1	-	-	-	=	=	=	1	-	-	3	1	1
***Ireland***	1	1	1	-	-	-	3	2	2	1	1	1	5	4	4
***Italy***	2	2	2	1	-	1	-	-	-	1	1	1	4	3	4
***Latvia***	2	2	2	-	-	-	=	=	=	1	1	1	3	3	3
***Lithuania***	1	1	1	1	-	-	=	=	=	-	-	-	2	1	1
***Luxembourg***	1	-	1	-	-	-	1	1	1	1	1	1	3	2	3
***Malta***	-	-	-	-	-	-	1	1	1	1	1	1	3*	3*	3*
***The Netherlands***	1	1	1	-	-	-	-	-	-	2	2	2	3	3	3
***Poland***	1	1	1	1	1	1	=	=	=	-	-	-	2	2	2
***Portugal***	-	-	-	-	-	-	1	1	1	1	1	1	2	2	2
***Romania***	2	2	2	1	1	1	1	1	1	1	1	1	5	5	5
***Slovakia***	1	1	1	1	-	1	=	=	=	-	-	-	2	1	2
***Slovenia***	1	1	1	1	1	1	=	=	=	-	-	-	2	2	2
***Spain***	-	-	-	1	1	1	1	1	1	-	-	-	2	2	2
***Sweden***	1	1	1	-	-	-	1	1	1	-	-	-	2	2	2
**TOTAL**	29	25	27	13	10	12	18	17	16	16	13	12	77	66	68

Individuals in a leadership position of a national genetics or oncology society but without clinical qualifications were counted in the respective genetics or oncology professional group. Of the 29 clinical/medical geneticists participating in Phase I, 13 were in leadership positions of their national clinical/medical genetics society. Of the 13 clinical/medical oncologists participating in Phase I, 6 were in leadership positions of their national clinical/medical oncology society. * - final numbers include the participation of an otherwise qualified individual. ‘-’ designates that an invitation was sent but no response was received. ‘=’designates that no invitation was sent due to genetic counsellors not being active in the Member State according to the most recent available data.^12^

### Phase I

**Fig. 1:**
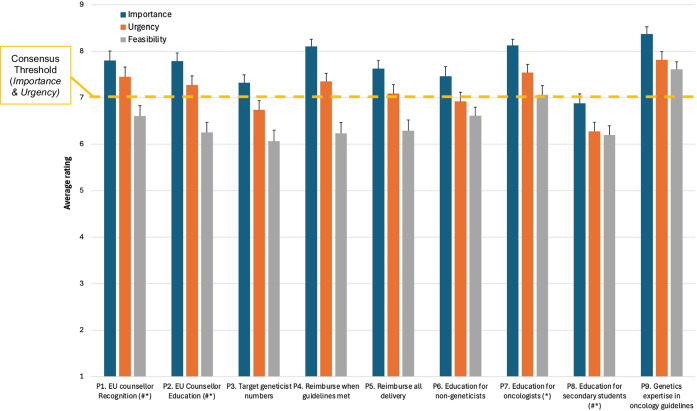
Average Importance, Urgency and Feasibility ratings from *n* = 77 participants for strategies proposed in Phase I. Error bars represent standard error. Consensus Importance and Urgency was defined a priori as an average rating ≥7. # - significant differences between professional groups, Importance rating (*p* < 0.05). * - significant differences between professional groups, Urgency rating (*p* < 0.05).

Six out of nine strategies met consensus criteria (average rating ≥7 out of 9) for both Importance and Urgency, with another two strategies meeting consensus criteria for Importance only (Fig. 1). Average ratings were higher for Importance than Urgency across all strategies. Significant differences in ratings between professional stakeholder groups were found for strategies P1, P2, P7, and P8 (8.7≤χ^2^(3)≤16.6; *p* < 0.05). Post-hoc pairwise comparisons showed significantly higher Importance and Urgency ratings of strategies P1 (genetic counsellor recognition) and P2 (genetic counsellor education/accreditation) by Genetic Counsellors compared to Clinical/Medical Geneticists and, to a lesser extent, Clinical/Medical Oncologists (*p* < 0.02; Table 3). Additionally, Cancer Patient Organisation representatives rated genetics education programs for secondary students (P8) as being significantly more Important and Urgent than genetic counsellors (*p* < 0.03).

**Table 3:** Median (interquartile range) Importance, Urgency, and Feasibility ratings for each strategy, stratified by professional group.

Strategy Number	Clinical / Medical Geneticists			Clinical / Medical Oncologists			Genetic Counsellors			Cancer Patient Representatives		
	*Import*.	*Urgency*	*Feasib*.	*Import*.	*Urgency*	*Feasib*.	*Import*.	*Urgency*	*Feasib*.	*Import*.	*Urgency*	*Feasib*.
P1^#*^	7 (4)^$^	7 (3)^$^	6 (3)	8 (1)	8 (3)^$^	8 (2)	9 (0)^~^	9 (0)^~!^	7 (3)	9 (1)	7 (2)	8 (3)
P2^#*^	7 (2)^$^	6 (3)^$^	6 (2)	8 (2)	7 (4)	7 (2)	9 (0)^~^	9 (1)^~^	7 (4)	9 (1)	7 (2)	7 (2)
P3	7 (2)	7 (3)	7 (4)	7 (1)	7 (2)	6 (2)	7 (2.5)	7 (4)	5 (2)	9 (2.5)	8 (3)	6 (3.5)
P4	8 (2)	7 (4)	7 (3)	9 (1)	8 (2)	7 (2)	9 (0.5)	7 (3)	6 (2)	9 (0)	9 (2.5)	7 (4)
P5	8 (3)	7 (3)	7 (3)	8 (2)	7 (2)	7 (3)	9 (2.5)	7 (3)	6 (2.5)	9 (2.5)	8 (3)	7 (3)
P6	8 (2)	7 (3)	7 (3)	7 (3)	7 (2)	6 (2)	8 (2.5)	7 (2.5)	6 (2)	9 (1.5)	8 (3)	8 (3)
P7^*^	9 (1)	8 (2)	7 (2)	8 (2)	7 (3)	7 (3)	9 (2)	7 (2)	7 (2)	9 (0)	9 (1.5)	8 (3)
P8^#*^	7 (2)	7 (3)	6 (2)	7 (3)	6 (2)	6 (2)	6 (2)^%^	5 (1)^%^	6 (1)	8 (2)^$^	7 (1.5)^$^	7 (3)
P9	9 (1)	8 (2)	8 (2)	9 (1)	8 (3)	8 (2)	9 (1)	8 (3)	7 (2)	9 (0)	9 (0.5)	9 (1.5)
A1^#^	8 (2)	7 (3)	7 (3)	7 (4)	5 (6)	6 (4)	6 (2)^%^	6 (3.5)	7 (2.5)	9 (2)^$^	7 (2)	7 (4)
A2^#^	7 (4)^%^	6 (4)	6 (4)	7 (7)	5 (6)	7 (5)	6 (4)^%^	5 (3)	6 (3)	9 (3.5)^~$^	7 (3.5)	7 (3.5)
A3^*^^	8 (3)	8 (3)	6 (2)	6 (7)	6 (6)	5 (3)^%^	8 (4)	7 (3)	6 (3)^%^	8 (2)	8 (2.5)	8 (3)^!$^
A4^#*^^	8 (2)	7 (2)	7 (2)	5 (7)^%^	5 (6)^%^	5 (4)^%^	8 (2)	7 (2)	7 (2)	9 (2)^!^	9 (4.5)^!^	9 (3)^!^
A5^*^^	8 (3)	7 (3)	7 (4)	7 (6)	5 (6)^%^	6 (4)	8 (4)	8 (4)	6 (2.5)^%^	9 (2)	9 (3)^!^	8 (2.5)^$^
A6	8 (1)	7 (3)	7 (2)	7 (7)	5 (6)	6 (4)	7 (2.5)	7 (2.5)	7 (3.5)	8 (4)	7 (4)	8 (4.5)
A7	8 (5)	8 (4)	6 (3)	8 (4)	6 (4)	7 (3)	8 (2)	8 (2.5)	7 (3)	7 (3)	6 (3.5)	6 (3)
A8^*^	7 (2)	7 (3)	7 (2)	7 (7)	5 (6)^$^	6 (2)	9 (2)	9 (1.5)^!^	7 (3.5)	9 (3)	8 (4)	7 (3)
A9^#^^	7 (8)	6 (7)	5 (6)	6 (8)	5 (7)	5 (7)	6 (6)	6 (7)	5 (4.5)	9 (1.5)	7 (5)	7 (6.5)
A10^#*^^	5 (4)^%^	5 (3)^%^	5 (4)^%^	5 (4)^%^	5 (4)^%^	4 (2)^%^	7 (1)	6 (2)	5 (3.5)	9 (2)^~!^	8 (3)^~!^	8 (4.5)^~!^

# - significant main effect of stakeholder group on Importance ratings. * - significant main effect of stakeholder group on Urgency ratings; ^ - significant main effect of stakeholder group on Feasibility ratings; ~ - significantly different from Clinical / Medical Geneticists in pairwise comparison (Bonferroni adjusted *p* < 0.05); ! – significantly different from Clinical / Medical Oncologists in pairwise comparison (Bonferroni adjusted *p* < 0.05); $ - significantly different from Genetic counsellors in pairwise comparison (Bonferroni adjusted *p* < 0.05); % - significantly different from Cancer Patient Representatives in pairwise comparison (Bonferroni adjusted *p* < 0.05).

### Phase II

A total of 10 additional strategies (Table 4) were aggregated from 64 total proposals from 30 Delphi participants (*13 clinical/medical geneticists; 1 clinical/medical oncologist; 9 genetic counsellors; 6 cancer patient organisation representatives; 1 otherwise qualified expert*) in Phase I and presented to participants for rating in Phase II.

**Table 4:** Additional European strategies for addressing common barriers to genetic counselling aggregated from 64 individual strategies proposed in Phase I.

Strategy Number	Descriptive Text
A1	EU support for the creation and hosting of online genetic counselling educational materials to ***support*** counselling delivered face-to-face or by phone/video.
A2	EU support for the creation and hosting of online genetic counselling materials aiming to ***provide*** counselling in common pre- and/or post-test scenarios.
A3	EU-wide assessment of barriers and approaches to increasing the attractiveness of health professions and the retention of health professionals, both generally and specific to genetics.
A4	EU-led campaign to promote general genetics knowledge and awareness of genetic cancer/disease predispositions in the population.
A5	EU support (financial and/or regulatory) for generalised genetic counselling training and recognition pathways for existing health professionals (*e.g. nurses, non-geneticist physicians, molecular biologists/pathologists*).
A6	EU support for the creation and hosting of digital tools capable of securely and efficiently collecting family history and other cancer risk data from patients.
A7	EU regulation of direct-to-consumer genetic testing.
A8	Dedicated EU funding to support new genetic counselling training programs in Member States (*e.g. continuing education for non-genetics health professionals; degree programs for genetic counsellors*).
A9	EU-wide restriction of germline genetic testing to academic centres.
A10	Establish a European genetic literacy institute to lead the development of European initiatives.

Strategies aggregated from greater numbers of individual inputs are listed first (e.g. the greatest number of individuals (*n* = 7) independently proposed strategies related to online educational genetic counselling materials, so aggregated strategies on this topic are listed first).

**Fig. 2:**
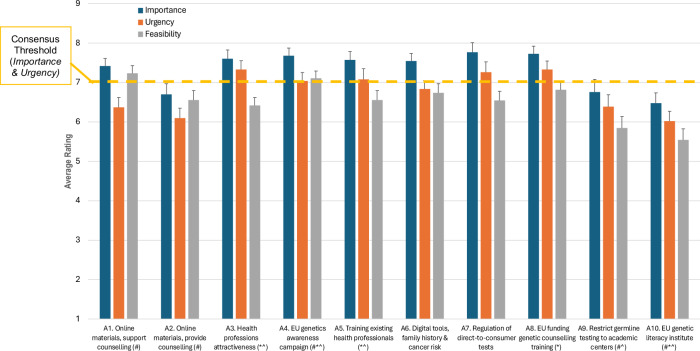
Average Importance, Urgency and Feasibility ratings for additional strategies aggregated from participant proposals in Phase I; these aggregated solutions were rated by *n* = 66 participants in Phase II. Error bars represent standard error. Consensus Importance and Urgency was defined a priori as an average rating ≥7. # - significant differences between professional groups, Importance rating (*p* < 0.05). * - significant differences between professional groups, Urgency rating (*p* < 0.05). ^ - significant differences between professional groups, Feasibility rating (*p* < 0.05).

Importance and Urgency ratings for five out of ten aggregated strategies met consensus criteria (average rating ≥7 out of 9); Importance but not Urgency ratings met consensus criteria for two additional aggregated strategies (Fig. 2). Average importance ratings were higher for Importance than Urgency across all strategies.

Cancer patient organisation representatives were overall the most positive regarding the Importance, Urgency, and Feasibility of Aggregated solutions – significant main effects of professional stakeholder group for strategies A1, A2, A3, A4, A5, A8, and A10 (8.8≤χ^2^(3)≤24.1; *p* < 0.04) were mostly driven by the more positive ratings of cancer patient organisation representatives compared to one or more professional groups (*p* < 0.05; Table 3). The exception is strategy A8 related to EU funding for genetic counselling training, which genetic counsellors rated as being significantly more Urgent than clinical/medical geneticists (*p* < *0.04*).

No single strategy received priority votes from a majority of Delphi participants. However, five strategies received a clear plurality of priority rankings, ranked as Priority #1, #2 or #3 by at least 15 participants (weighted Borda count range: 32 – 67): P1, P2, P4, P7, P9. All other strategies received Priority rankings from 8 or fewer participants (weighted Borda count ≤ 15).

### Phase III

**Fig. 3:**
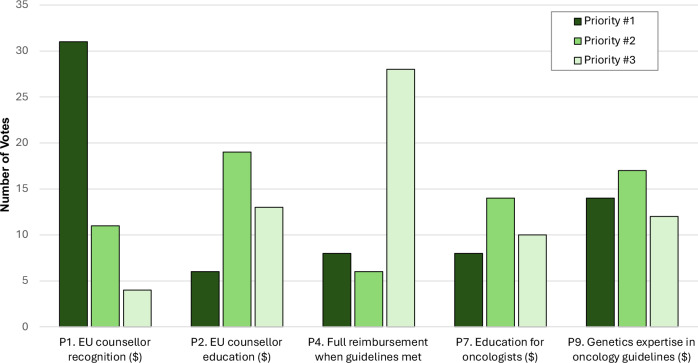
Number of Phase III priority votes for the five strategies which received a plurality of priority rankings in Phase II (68 voters). $ - significant differences in prioritisation between professional groups (*p* < 0.05). Weighted Borda count totals corresponding to vote totals for each strategy are as follows (maximum possible score = 204): P1 = 119; P2 = 69; P4 = 64; P7 = 62; P9 = 88.

Priority rankings for the five strategies receiving a plurality of Phase II priority rankings are displayed in Fig. 3. A statistically significant difference in priority rankings between these five strategies was observed (main effect: χ^2^(4) = 18.611, *p* < 0.001). Post-hoc tests showed that strategy P1 (EU genetic counsellor recognition) received significantly higher weighted rankings than strategies P2 (EU-wide genetic counsellor education/accreditation; *p* = 0.03), P4 (full reimbursement when guidelines are met; *p* = 0.02), and P7 (genetics continuing education for oncologists; *p* = 0.01). No other post-hoc differences between priority rankings of strategies were found (*p* > 0.34).

Significant differences in priority rankings between professional stakeholder groups were present for strategies P1, P2, P7, and P9 (genetics expertise in oncology guideline development)(13.1≤χ^2^(3)≤19.2; *p* < 0.01). Post hoc tests revealed that this significant main effect was driven for all four strategies by significant differences in the priority rankings of genetic counsellors compared to other professional groups: higher prioritisation of strategy P1 compared to all other groups (*p* = 0.02); higher prioritisation of strategy P2 compared to geneticist and oncology physicians (*p* = 0.03); and a lower prioritisation of strategies P7 and P9 compared to geneticist physicians and patient organisation representatives (*p* < 0.04).

## Discussion

Integrating the perspectives of members of four key stakeholder groups including representation from all 27 EU Member States, this Delphi survey identified five priority European strategies for addressing common barriers to genetic counselling in cancer identified in preliminary work [3, 10]. All five priority strategies were judged by consensus to be both Important and Urgent and received a clear plurality of priority rankings amongst 19 evaluated strategies. Of these five priority strategies, strategy P1 – *EU-wide recognition of genetic counsellors as allied health professionals and appropriately qualified experts to deliver genetic counselling* – was ranked as highest priority, albeit with notable differences in its relative prioritisation across stakeholder groups. Similar differences in relative prioritisation, as well as Importance and Urgency ratings, were noted for most priority strategies, with implications for future implementation. Individual discussion of each priority strategy follows in order of relative prioritisation (*Phase III Borda count*) below, with a focus on implementation pathways and potential hurdles.

*Strategy P1 – EU-wide recognition of genetic counsellors as allied health professionals and appropriately qualified experts to deliver genetic counselling (Moderate Feasibility: 6.6/9 in Phase I)*

Recognition of genetic counsellors throughout EU Member States has been proposed previously, with work in this area currently underway within the European Board of Medical Genetics, Genetic Nurse and Genetic Counsellor Branch [17]. Genetic counsellors, typically Master’s degree qualified allied health professionals, have been demonstrated to deliver quality genetic counselling services in multiple clinical scenarios, and are recognised and well-integrated into health systems in France and several countries outside the EU, e.g. Australia, the United Kingdom, and the United States [18]. An estimated 570 trained genetic counsellors currently exist in Europe [12]. However, their integration into the delivery of genetic services in the EU is limited by a lack of recognition of genetic counselling as an independent health profession in all Member States except France [3]. An estimated 340 of these 570 genetic counsellors (60%) are present in 14 EU countries outside France; recognition thus presents a first step towards addressing immediate genetic counselling workforce capacity barriers [12].

Implementing genetic counsellor recognition across the EU will require broad support from, in particular, clinical/medical geneticists – recent positive experiences in Iceland and Malaysia and challenges in Malta related to professional genetic counsellor recognition within the health systems have noted that the support, or lack thereof, of geneticist physicians has been decisive [19, 20]. Additional support from cancer patient organisations can provide further momentum.

Significant differences in stakeholder perspectives in this Delphi survey indicate that additional dialogues between clinical/medical geneticists and genetic counsellors are needed to build a broad mandate for timely genetic counsellor recognition; considering only the responses of clinical/medical geneticists, Importance (7.2/9) but not Urgency (6.9/9) ratings would have met consensus criteria. The reasons underpinning these discrepant perspectives were not captured in this Delphi study. However, and encouragingly, dialogues between genetic counsellors and clinical/medical geneticists have recently advanced genetic counsellor recognition in German-speaking countries [21]. The European Board of Medical Genetics and European Society of Human Genetics present appropriate forums for such dialogue on a European level, as both organisations already integrate both clinical/medical geneticists and genetic counsellors into their membership. The upcoming EU Joint Action on Personalised Cancer Medicine includes support for such dialogues within its project objectives. These upcoming European dialogues can then provide context and momentum for national discussions critical to furthering recognition efforts in relevant Member States.

*Strategy P9 – Mandatory inclusion of genetics expertise in the creation and revision of national/EU clinical oncology guidelines (High Feasibility: 7.6/9 in Phase I)*

Including a genetics expert in the process of creating and revising national and European oncology guidelines presents a straightforward solution rated as being highly Important, Urgent, and Feasible across stakeholder groups. Several Delphi survey participants noted that genetics expertise is already included in national oncology guideline processes in their Member States. An EU-wide mandate would expand these practices to help facilitate equitable integration of genetics into oncology practice across Member States.

Immediate next steps towards implementation are planned within the upcoming EU Joint Action on Personalised Cancer Medicine – an initial mapping of the present inclusion of genetics expertise in the creation and revision of national oncology guidelines across Member States, followed by direct dialogue with the relevant national health authorities and/or oncology societies to facilitate procedural updates and explore the possibilities for such a ‘hard’ or ‘soft’ EU mandate.

*Strategy P2 – Establish an EU system of registration, education and accreditation for genetic counsellors (allied health professional) with legal weight in Member States (Moderate Feasibility: 6.3/9 in Phase I)*

Our preliminary work noted concerns related to the high burden of establishing national registration/accreditation and education frameworks to serve low numbers of genetic counsellors within a given Member State [3]. While understandable, this also leads to a genetic workforce ‘chicken and egg’ problem, wherein low numbers of genetic counsellors preclude the establishment of national registration/accreditation/education frameworks, but the absence of these frameworks inhibits the training and registration of greater numbers of genetic counsellors.

Accordingly, a distributed system for ensuring adequate training and practice standards across Member States is envisioned as a means of supporting increased integration of genetic counsellors without disproportionate national burdens. Existing EU regulations provide guidance on how such a distributed system could function. Directly establishing a supra-national mandatory European system for the education, registration, and accreditation of genetic counsellors is likely a legal non-starter, given that primary responsibility for organising and delivering medical care and health services is explicitly the domain of Member States [22].

However, once the genetic counselling profession is officially recognised across multiple Member States (*i.e Strategy P1 as an essential first step*), the existing Directive 2005/36/EU would permit genetic counselling qualifications to be immediately transferred across these Member States through the ‘general system’ for recognition of qualifications. This ability to transfer qualifications across borders would de facto support a distributed system for genetic counsellor education; such a system will be particularly critical to support the initial growth of the recognised profession across the EU, as Masters programs accredited by the European Board of Medical Genetics are presently only available in France, Spain, Portugal, Italy, and Austria. A collaborative Nordic Masters program is also planned to undergo accreditation procedures and start accepting students in 2027. Key to ensuring the smooth operation of a distributed education system will be the continued standardisation of Masters of Genetic Counselling qualifications – e.g. according to the core curriculum established by the European Board of Medical Genetics, Genetic Nurse and Genetic Counsellor Branch [23] – to simplify mutual recognition procedures. National genetic counsellor registration procedures should be similarly standardised to support reciprocal arrangements between Member States and international mobility; here the voluntary European registration system supported by the Genetic Nurse and Genetic Counsellor Branch also already provides a framework [11].

Should growth of the recognised genetic counsellor profession follow strong patterns seen in Australia and the United States [12], the development of additional Masters programs will rapidly become more financially viable for universities across the EU. In this case, care will need to be taken to ensure sufficient educational opportunities are developed in Eastern European countries to support equitable expertise distribution across Member States. Additionally, proportional increases in available genetic counselling jobs will be required across Member States to realise the benefits of increased European genetic counselling training capabilities within the healthcare workforce. Over the longer-term, genetic counsellors could aim to eventually join streamlined ‘automatic recognition’ procedures under EU Directive 2005/36/EU currently available to seven established ‘sectoral professions’ to further promote mobility: medical doctors, nurses, midwives, dental practitioners, pharmacists, architects, and veterinary surgeons [24].

*Strategy P4 – Mandate full reimbursement of genetic counselling when conducted according to national or European guidelines (Moderate Feasibility: 6.2/9 in Phase I)*

Our preliminary work noted that reimbursement of/payment for genetic counselling services was at least partly limited for health practitioners and/or patients in 13 of 27 Member States broadly distributed across Southern, Eastern, and Western European regions [3]. This is despite the widespread inclusion of genetic counselling in clinical practice guidelines and national legislation – in several Member States, incomplete reimbursement for counselling persists despite the existence of legal mandates for pre- and/or post-test genetic counselling [3]. Such reimbursement challenges are hardly new, as a European Society of Human Genetics Public and Professional Policy Committee workshop in the year 2000 also emphasised ‘financial coverage’ as being a key issue impacting genetic service provision [25].

Prior study suggests that the unclear economic utility of genetic counselling is likely at least partly responsible for ongoing reimbursement challenges [26], with further work needed to ensure the universal inclusion of genetic counselling within emerging personalised medicine reimbursement models [27]. Accordingly, while a European reimbursement mandate promises to address a key genetic counselling barrier, the broad uptake of such a mandate across Member States would be likely predicated on clear demonstrations of long-term economic utility and sustainability. A 2022 review of economic evaluations of genomic/genetic tests and related genetic counselling notes that the evidence base upon which economic analyses can be conducted is presently ‘under-developed’ [28]. A planned multi-country cost-effectiveness analysis of genetic counselling in cancer prevention and care contexts in the upcoming EU Joint Action on Personalised Cancer Medicine will provide a first step towards closing this evidence gap.

*Strategy P7 – Mandatory education for clinical oncologists, continuing and as a part of fellowship training, regarding screening for cancer predisposition syndromes (e.g. when to refer, how to screen) (High Feasibility: 7.1/9 in Phase I)*

Global recommendations for oncology fellowship curricula from the European Society for Medical Oncology (ESMO) and American Society for Clinical Oncology (ASCO) have noted that genetics competencies are required to deliver best-practice medical oncology since 2016 [29]. Further, the most recent (2023) recommendations include an entire section dedicated to competencies related to genetic testing and counselling [30]. However, both our prior work and the results of this Delphi survey indicate that present educational programs for medical oncologists do not sufficiently support the acquisition of these competencies [3].

Tailored education specific to the needs of oncologists – i.e. as compared to generalised training in cancer predisposition syndromes – best supports genetics competency acquisition and integration [31]. However, the availability of such tailored education is presently sparse [32], including 2-day courses delivered by the European Society for Paediatric Oncology, as well as ESMO in conjunction with the European Reference Network on Genetic Tumour Risk Syndromes (ERN GENTURIS).

Further, the most recent available data indicate that ESMO/ASCO curricular recommendations are presently only fully implemented in fellowship training in four Member States, with these recommendations noted as being ‘adapted’ or ‘partially integrated’ in a further 11 Member States [30]. Whether and how genetics information is included in fellowship training in circumstances were ESMO/ASCO recommendations are adapted, partially integrated, or not observed has not been chronicled.

Recently started and upcoming EU projects present first steps towards enhancing the availability of educational opportunities: the Joint Action on Networks of Expertise in Cancer (‘JANE-2’); Joint Action EUnetCCC (Comprehensive Cancer Centers); and Joint Action on Personalised Cancer Medicine. These Joint Actions plan to map the current inclusion of genetics in core education, fellowship training, and continuing education of relevant physicians and health professionals, including but not limited to oncologists; genetic literacy needs are, of course, not constrained to oncologists but also include the full breadth of practitioners involved in cancer care pathways, e.g., surgeons, gynaecologists, general practitioners, nurses. Based on identified gaps, new educational content and/or courses will be developed for inclusion in general medical education, fellowship/residency, and continuing education programmes, in collaboration with European and national stakeholders.

### Limitations

The robust size and multistakeholder composition of our Delphi survey cohort is one of its strengths – our cohort of 77 participants is in line with recommended sample sizes for ‘highly replicable’ results from multistakeholder Delphi surveys [33]. However, the potential impact of an unequal distribution of participants across stakeholder groups on results should be noted, as well as geographic asymmetries. While unsurprising given the topic area, the relative over-representation of clinical/medical geneticists compared, in particular, to clinical/medical oncologists emphasises the need for further interdisciplinary dialogue to guide the broad implementation and, where necessary, adaptation of identified strategies. Such dialogue can help account for perspectives which may not have been represented in this Delphi survey. Similar sensitivities should also be integrated into interdisciplinary dialogues related to the implementation of priority strategies at the national level – while all Member States were represented in our Delphi cohort, not all stakeholder groups were represented from each Member State.

## Summary and conclusions

The five priority strategies identified present a clear strategic roadmap to address common European barriers to genetic counselling in cancer care. Upcoming and recently started European projects provide opportunities to push these priorities toward implementation, in collaboration with national and European cancer patient organisations and genetics and oncology societies.

## Data Availability

The de-identified data presented in this manuscript are available upon reasonable request.
